# Draft genome sequence of *Enterobacter asburiae* strain P67 isolated from fecal specimen of a *Helicobacter pylori*-infected male patient of Sikkim, India

**DOI:** 10.1128/mra.01065-25

**Published:** 2025-11-24

**Authors:** Srichandan Padhi, Puja Sarkar, Tara Sharma, Arjan Narbad, Amit Kumar Rai

**Affiliations:** 1 University Centre for Research and Development, Chandigarh University 127373 , Mohali, India; 2 BRIC-National Agri-Food and Biomanufacturing Institute (BRIC-NABI), Mohali, India; 3 Department of Microbiology, STNM Hospital 7308, Gangtok, India; 4 Quadram Institute Bioscience, Norwich Research Park Colney 7308, Norwich, England, United Kingdom; University of Pittsburgh School of Medicine, Pittsburgh, Pennsylvania, USA

**Keywords:** *Enterobacter asburiae*, antimicrobial resistance, *Helicobacter pylori*

## Abstract

We report here the draft genome sequence of an *Enterobacter asburiae* strain P67, sequence type 252 (ST252), isolated from a fecal specimen of a *Helicobacter pylori*-infected male patient from Sikkim, India. The genome was 4,731,308 bp, with G + C content of 55.53%, and contained genes related to antimicrobial resistance and virulence factors.

## ANNOUNCEMENT

*Enterobacter asburiae* is widely distributed as a gut commensal bacterium and also reported as an opportunistic pathogen causing nosocomial infections with high antimicrobial resistance and virulence features (1). An *E. asburiae* strain P67 was isolated on Brain Heart Infusion agar (supplemented with 10 µg/mL vancomycin, 10 µg/mL nystatin, 5 µg/mL cefsulodin, 5 µg/mL trimethoprim, 8 µg/mL amphotericin B, and 2.5 U/mL polymyxin B) from a fecal specimen of an *H. pylori*-infected male patient from Sikkim, India. The plates were kept at 37°C for 4–5 days under anaerobic conditions. The ethical clearance was obtained from the Institutional Ethical Committee, STNM Multispeciality Hospital (STNMH), Sikkim (Memo no. OS/IEC/STNMH/22, dated 22 April 2022). Genomic DNA was extracted using the Wizard Genomic DNA Purification Kit (Promega). DNA quantity was measured using Nanodrop 1000 (Thermo Fisher Scientific). The libraries were prepared using the Nextera XT DNA Library Preparation Kit (Illumina) and index primers (Illumina) and quantified using the Promega QuantiFluor dsDNA System (Catalog no. E2670). The final pool was double-SPRI size, selected between 0.5 and 0.7× bead volumes using sample purification beads (Illumina DNA Prep, [M] Tagmentation [96 Samples, IPB], 20060059), and analyzed with Qubit 3.0 and run Agilent Tapestation 4200. The pool was run at a final concentration of 1.5 pM on an Illumina NextSeq 500 using a Mid Output Flowcell (NSQ 500 Mid Output KT v2 [300 cycle] Illumina Catalog FC-404-2003) using a 2 × 150 paired-end protocol, generating 837,144 reads.

The reads were trimmed and quality checked on Fastp (v0.23.4, --length_required 36) (2) and FastQC (v0.12.1) (http://www.bioinformatics.babraham.ac.uk/projects/fastqc), respectively. Genome assembly was performed using SPAdes (v3.15.5) (3) and the quality of the assembly was measured using QUAST (v5.0.2) (4). The genome was annotated using the NCBI Prokaryotic Genome Annotation pipeline (5). All bioinformatics analyses were run with predefined settings, unless stated otherwise. Taxonomic assignment of the genome was performed using Type (Strain) Genome Server (6). ST was assigned using pubMLST (7). Antimicrobial-resistant (AMR) genes and virulence factors were predicted using CARD (8) and VFDB (9). The draft genome has 4,731,308 bp in a total of 42 contigs, with G + C content of 55.53%, *N*_50_ of 256,458 bp and *L*_50_ of 6. The genome consists of 4,574 genes, including 4,416 protein-coding genes, 69 tRNA, 5 rRNA genes, 78 pseudogenes, and 4 other genes. The strain was assigned to *E. asburiae* ST252. *In silico* analyses predicted multiple AMR genes (Table 1) and virulence factors (curli production assembly/transport protein [csgG], 23-dihydro-23-dihydroxybenzoate dehydrogenase [entA], and outer membrane protein A [ompA]).

**TABLE 1:** Antimicrobial resistance genes identified in *E. asburiae* strain P67

Gene	Class	Product
blaACT	Beta-lactam	Cephalosporin-hydrolyzing class C beta-lactamase ACT-3
qnrE	Quinolone	Quinolone resistance pentapeptide repeat protein QnrE4
Eclo_acrA	Cephalosporin/fluoroquinolone/glycylcycline/penam/phenicol/rifamycin/tetracycline	Resistance-nodulation-cell division (RND) antibiotic efflux pump
CRP	Fluoroquinolone/macrolide/penam	RND antibiotic efflux pump
fosA	Fosfomycin	Fosfomycin resistance glutathione transferase FosA_7
oqxB	Phenicol/quinolone	Multidrug efflux RND transporter permease subunit OqxB9
H-NS	Cephalosporin/cephamycin/fluoroquinolone/macrolide/penam/tetracycline	Major facilitator superfamily antibiotic efflux pump and RND antibiotic efflux pump

## Data Availability

This Whole-Genome Shotgun project has been deposited at DDBJ/ENA/GenBank with JBJIJW000000000. The version described in this paper is version JBJIJW010000000. The raw data at the SRA database can be accessed at SRS23212113.
